# Electrochemical Synthesis of Nitrite and Nitrate via Cathodic Oxygen Activation in Liquefied Ammonia

**DOI:** 10.1021/jacs.4c10279

**Published:** 2024-11-04

**Authors:** Moritz Lukas Krebs, Ferdi Schüth

**Affiliations:** Department of Heterogeneous Catalysis, Max-Planck-Institut für Kohlenforschung, Kaiser-Wilhelm-Platz 1, 45470 Mülheim, Germany

## Abstract

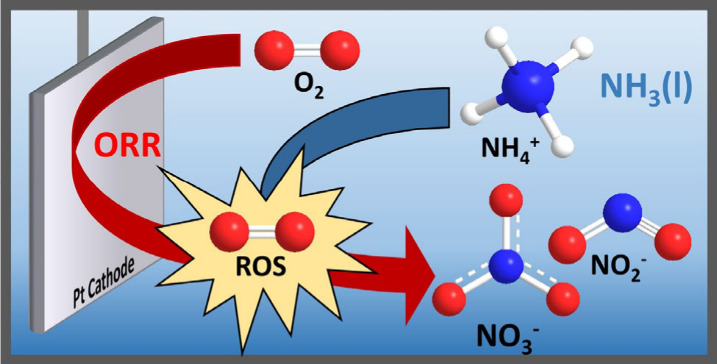

The electrochemical oxidation of ammonia (NH_3_) enables decentralized small-scale synthesis of nitrate (NO_3_^–^) and nitrite (NO_2_^–^) under ambient conditions by directly utilizing renewable energy. Yet, their electrosynthesis has been restricted to aqueous media and low ammonia concentrations. For the first time, we demonstrate here a strategy enabling the direct electrooxidation of liquefied NH_3_ to NO_3_^–^ and NO_2_^–^ by using molecular oxygen, achieving combined Faraday efficiencies above 40%.

Nitrite (NO_2_^–^) and nitrate (NO_3_^–^) are used for various industrial applications, including the synthesis of pharmaceuticals, nylon precursors, explosives, and fertilizer compounds.^1,2^ However, the conversion of ammonia to nitrogen oxides relies on the Ostwald process, which uses noble metal catalysts (Pt, Rh) at high temperatures (873–1073 K) and pressures (>400 kPa).^1^ In addition, this process also emits nitrous oxide (N_2_O), a greenhouse gas with a global warming potential 298 times that of CO_2_.^3^ Electrochemical ammonia oxidation to nitrogen oxides has emerged as a promising alternative, allowing direct utilization of renewable energy sources and enabling decentralized small-scale production of nitrogen oxides under mild conditions.^4−9^ Despite its potential, the electrochemical oxidation of ammonia to nitrogen oxides faces several challenges:

- Limited to aqueous systems with ammonia concentrations typically below 1 wt %.^10,11^
- Competing oxygen evolution reaction (OER) and nitrogen evolution reaction (NER).^6,12^
- Elevated overpotentials required for the activation of the oxygen source and subsequent oxidation steps.^11−16^
- Ammonia-induced corrosion of catalysts under applied bias.^16−18^

An unexplored yet promising concept involves the direct electrooxidation of liquefied ammonia gas (NH_3_(l)) with molecular oxygen. This approach could facilitate the synthesis of NO_2_^–^ and NO_3_^–^ at lower cell voltages without the limitation of low ammonia concentrations. Moreover, in a potential future with ammonia as a hydrogen carrier, NH_3_(l) would be the transport form and could thus directly be used as a raw material.

The electrochemical activation of O_2_ and synthesis of reactive oxygen species (ROS) via the oxygen reduction reaction (ORR) is feasible in many aprotic solvents.^19−22^ The formed superoxide radical anion (O_2_^•–^) can be protonated to form the hydroperoxyl radical (HO_2_^•–^), which is known to be a reactive oxidant.^23−26^ In the 1980s, pioneering research by Bard and coworkers demonstrated that the cathodic activation of O_2_ in NH_3_(l) on Pt electrodes is also possible. Irreversible oxygen reduction was observed in the presence of NH_4_^+^.^27^ Further mechanistic studies found that the ORR is facilitated in the presence of NH_4_^+^ ions, requiring significantly lower potential than the competing hydrogen evolution reaction (HER).^28,29^ We utilize the high overpotential of Pt electrodes in the HER to selectively form ROS via the competitive ORR in NH_3_(l) (Scheme 1).^28,30^ Moreover, we demonstrate that the so-formed ROS can in fact be used to synthesize NO_2_^–^/NO_3_^–^ directly in NH_3_(l). To the best of our knowledge, this is the first example of direct electrochemical nitrogen oxide synthesis in NH_3_(l).

**Scheme 1:**
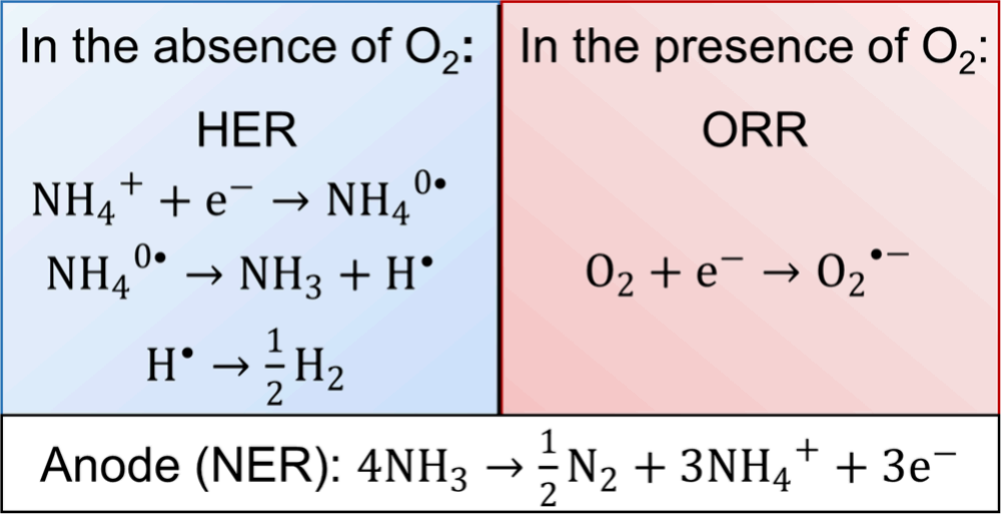
Reactions in NH_3_(l) in Both the Presence and Absence of Oxygen; HER Mechanism as Described by Little et al.^30^

To conduct experiments with NH_3_(l) under equilibrium conditions while providing additional oxygen partial pressures, we utilized an electrochemistry autoclave, which has been used in previous studies (see SI for details).^31,32^ In a typical experiment, the autoclave is assembled in a glovebox, filled with NH_3_(l) (30 mL), heated to 303 K, and then pressurized with ∼35 bar of a mixture of 4% O_2_ in Ar. This did result in an approximately 10 mM concentration of O_2_ dissolved in NH_3_(l).^33^ It is important to note that elevated oxygen contents might favor the formation of oxidized nitrogen species. However, we were cautious about the potential formation of explosive oxygen–hydrogen mixtures due to the cathodic HER. A more detailed description of the experiment design is outlined in the SI.

Cyclic voltammetry (CV) measurements in NH_3_(l) at 303 K were initially performed with an ammonium bromide (NH_4_Br) electrolyte (Figure 1a). The current response is significantly affected by the presence of oxygen. For 20 mA, a decrease in the cell voltage of ∼800 mV is observed, which is an expected result for the switch to ORR in NH_3_(l) on Pt electrodes.^28^ To further study the system, constant-current electrolysis was conducted at 20 mA until a total charge of 100 C was transferred (Figure 1b). It is evident that a decrease in the cell voltages is maintained during the measurements. However, a continuous increase in the voltage was observed, which we attribute to a decrease in the oxygen partial pressure due to the ORR. This is further supported by a steadily decreasing cell pressure (Figure S3). To confirm that the ORR has replaced the HER as the dominating cathode reaction, we analyzed the headspace atmosphere by gas chromatography (Figure 1c). In the absence of O_2_, the expected H_2_/N_2_ ratio of 3:1 is observed. Faraday efficiencies (FE) of ∼90% for H_2_ and N_2_ are measured, typical for ammonia decomposition in NH_3_(l).^30,34^ In the presence of oxygen, however, almost no H_2_ is found, indicating the absence of HER activity on the Pt electrode. Additionally, the FE for nitrogen formation remained constant, suggesting that the presence of oxygen does not directly interfere with the anode reaction.

**Figure 1:**
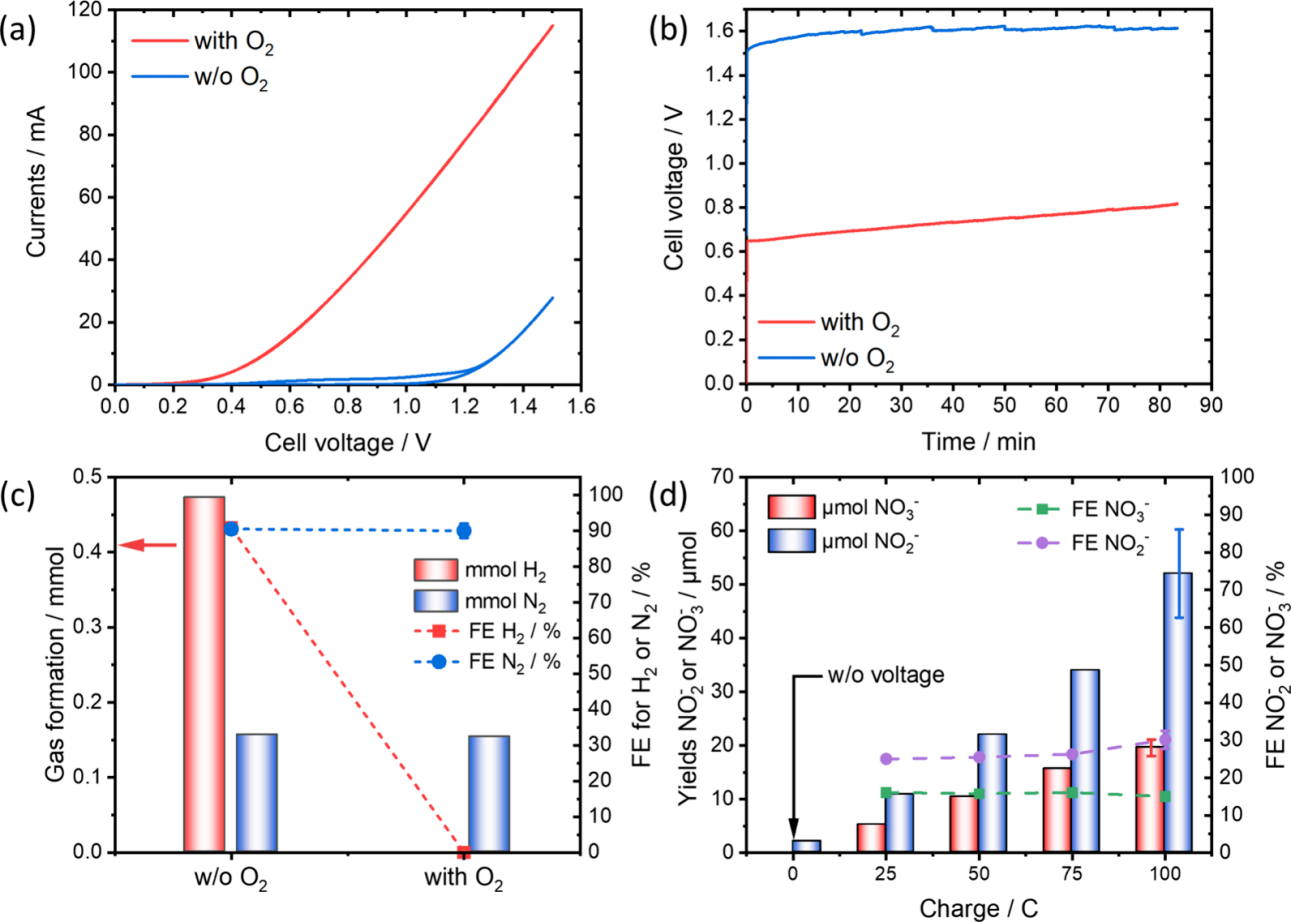
(a) CV scans in the presence of oxygen (red) and the absence of oxygen (blue) in the electrochemical cell. Measurements were conducted in NH_3_(l), using 1 M NH_4_Br as the electrolyte and a scan rate of 100 mV/s. (b) Corresponding electrolysis at a constant current of 20 mA. (c) Gas analysis performed after the reaction. (d) NO_2_^–^/NO_3_^–^ yields quantified by ion chromatography (IC). To measure nonfaradaic NO_2_^–^/NO_3_^–^ formation (results at 0 C), the cell was stirred for 3 h in the presence of oxygen without applying an external potential.

Ion chromatography (IC) was employed to quantify the produced NO_2_^–^/NO_3_^–^ species. As shown in Figure 1d, the IC confirmed the formation of NO_2_^–^/NO_3_^–^. The yields increased with the transferred charge. Specifically, 52 μmol (±8 μmol) of NO_2_^–^ and 20 μmol (±2 μmol) of NO_3_^–^ were produced after passing 100 C. To calculate the FE, we assumed theoretical oxidation of NH_3_ to the respective nitrogen oxides involving 6 e^–^ and 8 e^–^ for NO_2_^–^ and NO_3_^–^, respectively. Based on this assumption, corresponding FEs of 30% (±2%) for NO_2_^–^ and 15% (±1%) for NO_3_^–^ were determined. We emphasize that the FE reported serves as an additional theoretical descriptor, connecting the yields to the electrochemical energy input. To determine the actual FE, precise knowledge of the reaction mechanism is needed, which we are unable to provide due to the demanding reaction conditions. The nature of the oxidant has significant impact, introducing a hypothetical upper limit of 300% FE if O_2_^•–^ or HO_2_^•^ is the sole oxidant, as only 1 e^–^ is needed to form these potential 3e^–^ oxidants. A discussion of the potential reaction mechanism is outlined in Section 2.1 of the SI.

Significant nonfaradaic NO_2_^–^/NO_3_^–^ formation was ruled out by additional control experiments without applying an external voltage. Formation of gaseous nitrogen oxides was probed by absorbance FT-IR spectroscopy; however, no traces were detected (Figure S4). Experiments at various currents and pressures (Figures S5 and S6) show that fine-tuning reaction parameters improves FE for NO_2_^–^/NO_3_^–^. However, we kept a 20 mA current for all further experiments as the best balance between the yield and reaction time.

We further investigated the effect of varying reaction temperatures.^27−29^ Significantly increased yields were observed. Specifically, NO_2_^–^ yields of 123 and 139 μmol and NO_3_^–^ yields of 15 and 25 μmol at 323 and 343 K are achieved, respectively (Figure 2). These amounts of anions were formed rapidly due to the swift conversion of added oxygen, as evidenced by a sharp potential increase (Figure S7). The experiments concluded after 65 and 53 C of charge at 323 and 343 K, respectively. Consequently, a high FE for the NO_2_^–^/NO_3_^–^ synthesis was measured, with the NO_2_^–^ FE exceeding 100%. We also observed increased amounts of nonfaradaic NO_2_^–^/NO_3_^–^ formation (Figure S8). At 343 K, 19 μmol of NO_2_^–^ and 1 μmol of NO_3_^–^ are formed when stirring without an applied voltage for the same time. Still, the yields are significantly higher when an external voltage is applied, indicating that the stark increase in the FE is not solely attributed to nonfaradaic formation. Notably, even at elevated temperatures, no gaseous nitrogen oxides were detected (Figure S4).

**Figure 2:**
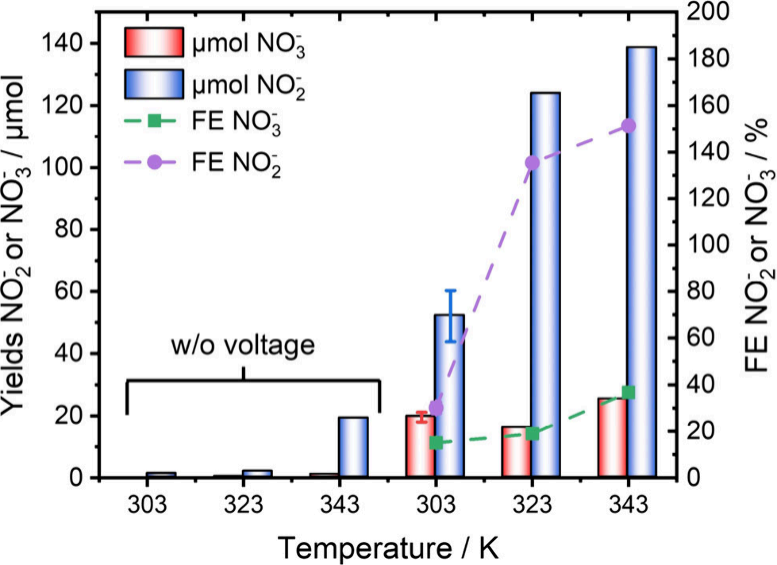
FE and NO_2_^–^/NO_3_^–^ yields were obtained at various temperatures using 1 M NH_4_Br as the electrolyte. In control experiments, solutions were stirred in the presence of oxygen for the same reaction time.

The formation of NO_2_^–^/NO_3_^–^ is likely influenced by the activity of the produced superoxides, which is modulated by the presence of protons.^28,29^ The presence of protons will likely result in the protonation to hydroperoxyl species, eventually oxidizing the ammonia to NO_2_^–^/NO_3_^–^ through a hydrogen atom transfer mechanism.^23,35,36^ Direct formation of the peroxide dianion (O_2_^2–^) and related peroxide species is also reported for NH_3_(l) but requires a significantly increased cathodic potential that depends on the presence/concentration of an additional proton source.^27^ Consistent with the findings, experiments varying the NH_4_Br concentration demonstrated increased NO_3_^–^ yields with an increasing ammonium salt concentration (Figure S9). Starting from 8% for a 0.2 M NH_4_Br electrolyte, the FE for NO_3_^–^ increased to 15% FE and 32% FE for 1.0 and 3.0 M electrolytes, respectively.

As the formed peroxide and hydroperoxyl species will eventually be reduced/disproportionate to H_2_O, the influence of H_2_O on the NO_2_^–^/NO_3_^–^ yields was studied. Although the cell voltage slightly increased with the addition of water (Figure S10), adding 1 mL of water resulted in an FE of ∼23% for NO_2_^–^ and ∼28% for NO_3_^–^, enhancing the cell efficiency and selectivity toward NO_3_^–^ (Figure 3). Additional experiments with H_2_O, but without gaseous O_2_ or in the absence of an external potential, showed only traces of NO_2_^–^, demonstrating that the role of H_2_O is limited to the superoxide pathway.

**Figure 3:**
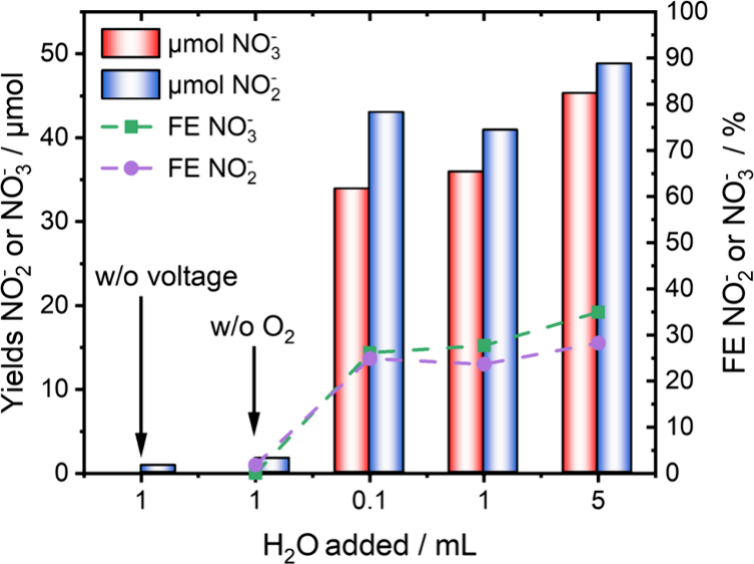
FE and NO_2_^–^/NO_3_^–^ yields at 303 K when water is added. Blank experiments without voltage were conducted by stirring the electrolyte in the presence of oxygen for 3 h.

The presence of a proton source thus significantly influences the yields of the NO_2_^–^/NO_3_^–^. However, the role of the ammonium ion (NH_4_^+^) still needs to be clarified. In order to explore this, the experiments described above were repeated in the presence of potassium bromide (KBr), excluding NH_4_^+^ (Figure S11). Regardless of the reaction conditions, such as the reaction temperature (Figure 4a) or the presence of water (Figure 4b), the overall yields for the NO_2_^–^/NO_3_^–^are substantially reduced. Notably, the formation of NO_3_^–^ was only observed at elevated temperatures (343 K) or with a significant addition of water (5 mL), resulting in FE values of 6% and 3%, respectively. Similarly, NO_2_^–^ yields were significantly reduced, with FE values as low as 6% for experiments conducted at 303 K.

**Figure 4:**
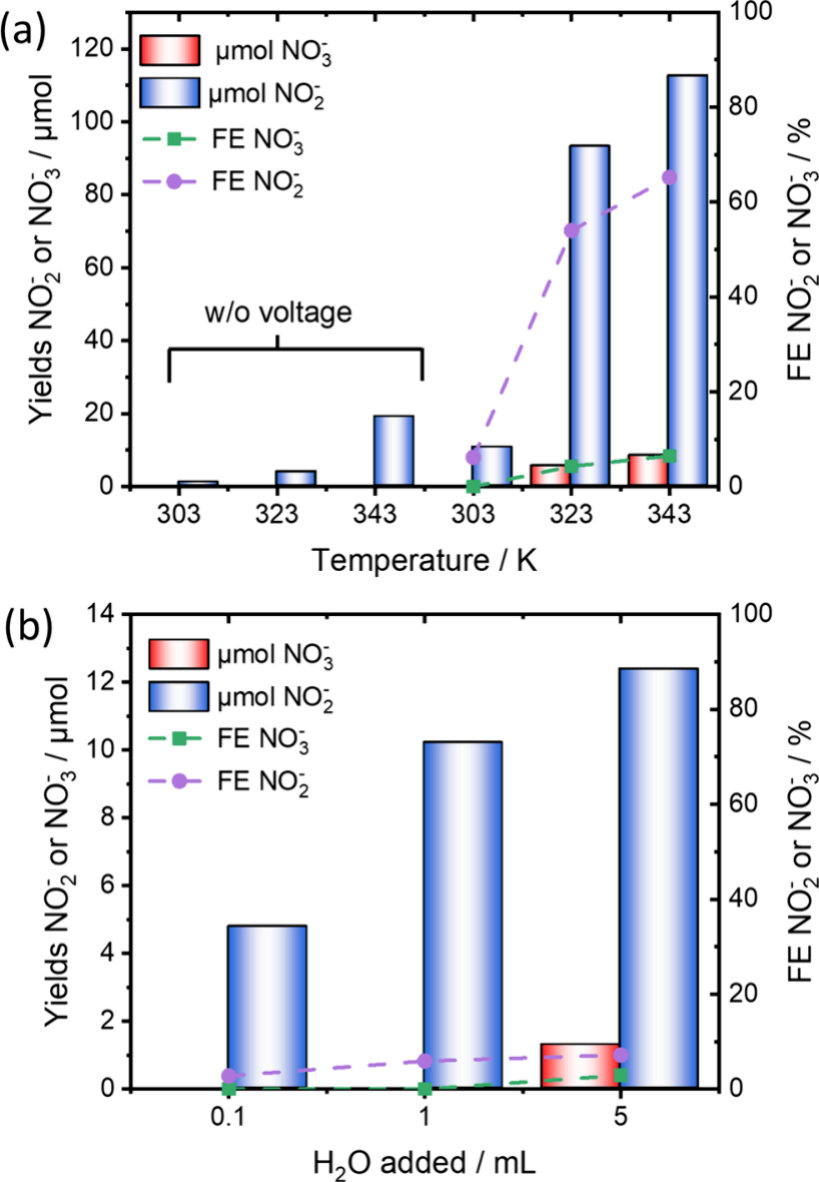
FE and yields for NO_2_^–^/NO_3_^–^ when KBr is used as the electrolyte. (a) Experiments at different reaction temperatures with and without applying an external potential. (b) Effect of water addition on the synthesis of NO_2_^–^/NO_3_^–^ at 303 K.

While the NO_2_^–^/NO_3_^–^ formation efficiency might be directly dependent on the presence or absence of NH_4_^+^, it is also known that potassium ions (K^+^) can lead to the precipitation of potassium superoxide (KO_2_) in NH_3_(l), potentially inhibiting the oxidation of the ammonia.^27^ Raman measurements after the reaction, however, failed to prove the existence of those species (Figure S12). Yet KO_2_ is a very volatile species, which makes isolation challenging. To determine if the formation of KO_2_ or the absence of NH_4_^+^ is responsible for the decreased yields, we extended our experiments to tetrabutylammonium bromide (TBAB, Figure S13). The reaction with TBAB led to only traces of NO_3_^–^ and a very low FE of 2% for the reaction with NO_2_^–^. Overall, the results suggest that the presence of NH_4_^+^ is of crucial importance for forming NO_2_^–^/NO_3_^–^, even in the presence of other, weaker proton donors, such as H_2_O. In fact, differences in the ORR mechanism are reported, depending on the reaction environment, e.g., the presence of different proton sources.^26,36^ Additional experiments have been performed with ammonium hexafluorophosphate, resulting in comparable results as for NH_4_Br (Figure S14), which, incidentally, also excludes the influence of the counteranion.

Lastly, experiments with potassium nitrite (KNO_2_) and potassium nitrate (KNO_3_) were conducted (Figure S15). Significantly increased NO_3_^–^ yields are detected when KBr is replaced by KNO_2_, suggesting that NO_2_^–^/NO_3_^–^ formation might not be independent of each other. Given that NO_2_^–^ can undergo oxidation in aqueous systems,^37^ we assume that both anodic and superoxide-mediated oxidation of the NO_2_^–^ may occur. Further mechanistic studies utilizing a new autoclave design are currently underway to clarify the oxidation pathway.

In conclusion, our experiments demonstrate that the electrochemical synthesis of NO_2_^–^/NO_3_^–^ from NH_3_ is not limited to aqueous systems but can be directly achieved in NH_3_(l). By exploiting the high overpotential of the HER in NH_3_(l), we present a strategy for selectively producing ROS via the ORR, which enables the oxidation of NH_3_ to both NO_2_^–^ and NO_3_^–^. Furthermore, we have shown that the presence and concentration of NH_4_^+^ is of significant importance for the NO_2_^–^/NO_3_^–^ formation. A pronounced effect was observed for the formation of NO_3_^–^, which is typically less accessible in the alkaline aqueous electrooxidation but is of greater interest than NO_2_^–^. Additionally, we explored different reaction conditions and found a positive correlation between the efficiency of NO_2_^–^/NO_3_^–^ formation and both the introduction of water and increased reaction temperatures.

## Supporting Information Available

Experimental details, information on the electro-autoclave and other equipment, chemicals, analytical methods, and detailed description of the procedure for catalytic experiments (PDF)
