# Supplementary material for: Electrochemical Synthesis of Nitrite and Nitrate via Cathodic Oxygen Activation in Liquefied Ammonia
Source: J Am Chem Soc. 2024 Nov 4;146(45):30753–7. doi: 10.1021/jacs.4c10279 (PMC11565706; doi:10.1021/jacs.4c10279)
Supplement: Supplementary file 1 — ja4c10279_si_001.pdf [file ja4c10279_si_001.pdf]

## Supporting Information for

# Electrochemical synthesis of nitrite and nitrate via cathodic oxygen activation in liquefied ammonia

Moritz Lukas Krebs[1]; Ferdi Schüth[1]*

[1]Department of Heterogeneous Catalysis, Max-Planck-Institut für Kohlenforschung, Kaiser-Wilhelm-Platz 1, 45470 Mülheim, Germany

*To whom correspondence should be addressed:

Email: schueth@kofo.mpg.de

**Table of Contents**


**1. Instruments and experimental procedures** ....................................................3
1.1 Chemicals and materials....................................................................................3
1.2 Electrochemistry autoclave ...............................................................................3
1.3 Electrochemical experiments ............................................................................5
1.4 Gas chromatography (GC).................................................................................6
1.5 Infrared spectroscopy (IR) .................................................................................6
1.6 Ion chromatography (IC) ...................................................................................6
1.7 Raman spectroscopy .........................................................................................7
1.8 X-Ray photoelectron spectroscopy (XPS) ........................................................7
1.9 Inductively coupled plasma-optical emission spectrometry (ICP-OES).........7
**2 Results** ................................................................................................8
2.1 Discussion on the reaction mechanism ............................................................8
2.2 Cell pressure.......................................................................................................10
2.3 Gas phase FT-IR spectra of the headspace ...................................................11
2.4 Yields in dependence of the applied currents .................................................12
2.5 Constant current electrolysis at different pressures.........................................13
2.6 Constant current electrolysis at different temperatures ..................................14
2.7 Non-faradaic formation of nitrite and nitrate.....................................................15
2.8 Experiments at different ammonium ion concentrations..................................16
2.9 Constant current electrolysis in the presence of water ....................................17
2.10 Constant current electrolysis for KBr electrolytes ...........................................18
2.11 Raman measurements......................................................................................19
2.12 Experiments with tetrabutylammonium bromide (TBAB) ................................20
2.13 Experiments with ammonium hexafluorophosphate ......................................21
2.14 Experiments in the presence of nitrite and nitrate salts ..................................22
2.15 Corrosion of the Pt anode ...............................................................................23
**3 References**..........................................................................................24

# 1. Instruments and experimental procedures

**<u>CAUTION!</u> MIXTURES OF OXYGEN, AMMONIA, AND HYDROGEN CAN BE EXPLOSIVE IF NOT DILUTED BY THE APPROPRIATE AMOUNT OF INERT GAS.**

## 1.1 Chemicals and materials

All chemicals were used as received without further purification unless otherwise specified. $HNO_3$ (J.T.Baker®; 65%), $H_2SO_4$ (J.T.Baker®; 95.0 - 98.0%), KBr (Sigma-Aldrich;), $KNO_3$ (Sigma-Aldrich; 99.995 %), $KNO_2$ (Sigma-Aldrich; ACS reagent, ≥96.0%), $KO_2$ (abcr; 96%), $NH_4Br$ (Sigma-Aldrich; ACS reagent > 99 %), $NH_4NO_3$ (Sigma-Aldrich, for analysis EMSURE® ACS), $NH_4PF_6$ (TCI; > 98 %), Pb(II)-acetate trihydrate (Sigma Aldrich; ACS reagent, 99 %), Pt foil (Goodfellow GmbH; d = 0.05 mm, 99.95%), TBAB (Sigma Aldrich; >98% ACS). BDD anodes, DIACHEM® Electrode (Typ 52) were purchased from CONDIAS, Itzehoe / Germany.

To remove excess water, all electrolyte salts (KBr, $KNO_3$, $KNO_2$, $NH_4Br$, $NH_4NO_3$) were ground into a fine powder (if necessary) and dried in a vacuum oven at 323 K overnight. Subsequently, stable salts underwent an additional drying step under high vacuum ($10^{-5}$ mbar) at 393 K ($NH_4Br$) or 423 K ($KNO_3$, $KNO_2$, KBr,) for at least 12 h. After the drying step(s), the dried salts were stored in a glovebox to prevent moisture uptake.

Milli-Q (MQ) water (18.2 MΩ) was used for all of the experiments and for the clean-up.

All common gases were received from Air Liquide. A mixture of 4% $O_2$ in Ar was prepared for experiments requiring the presence of oxygen.

## 1.2 Electrochemistry autoclave

The electrochemistry autoclave, designed and built in-house (Figures S1-2), is capable of sustaining temperatures up to 473 K and pressures up to 100 bar.[1,2] It is equipped with a JUMO dTrans p30 pressure gauge and a type K thermocouple protected by a Teflon hose. The exterior of the autoclave is made of stainless steel (1.4571), while the interior is protected by a Teflon lining. The sealing materials are made of PEEK and Teflon. Platinum wires are used to provide electrical contact. One wire forms a coil ($A_{geo}$ ~ 14 $cm^2$), while the other provides an electrical connection to a clamping device, which can be used to equip the autoclave with different electrodes. In this study, the electrode was equipped with an electrode holder to house a platinum foil (1 cm x 1 cm). A third platinum wire can be used as a pseudo-reference electrode; however, this option was not utilized in this study.

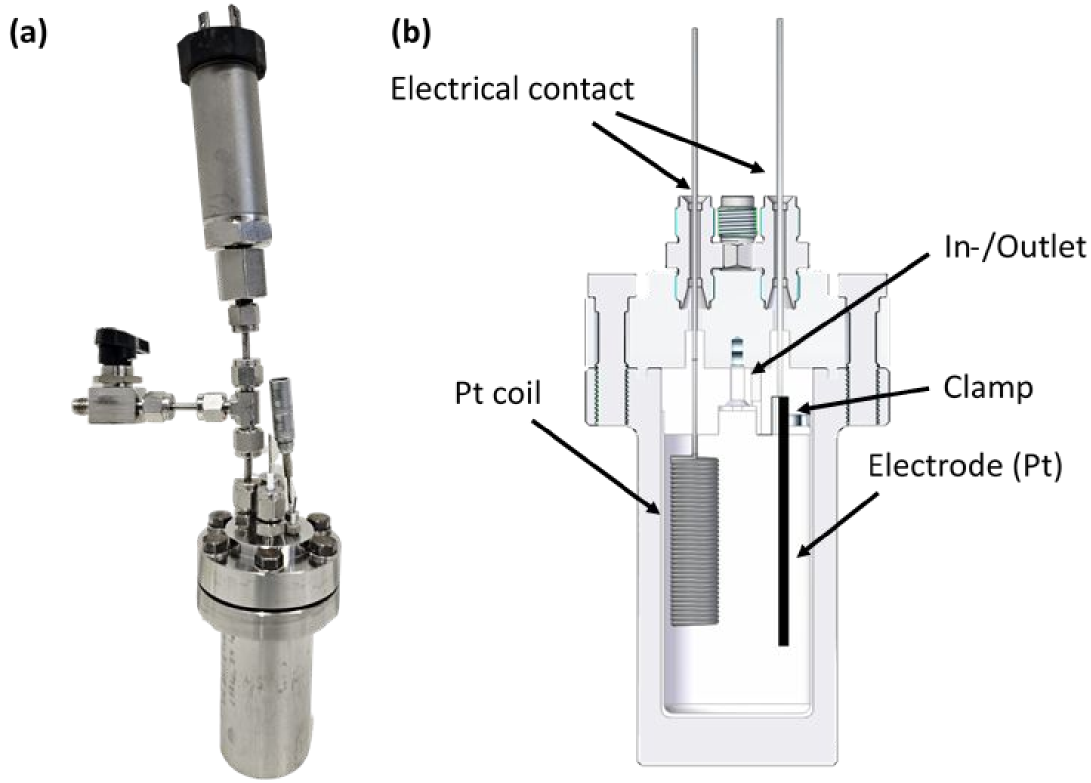


***Figure S1:*** *a) Picture of the closed electrochemistry autoclave for experiments at up to 473 K and 100 bar pressure. b) Drawing of the used high-pressure, high-temperature electrochemistry autoclave as lateral cut. In all reactions, a Pt coil was used as the cathode, while a Pt sheet (1 cm x 1 cm; simplified as a black bar) was used as the anode in the two-electrode configuration.*

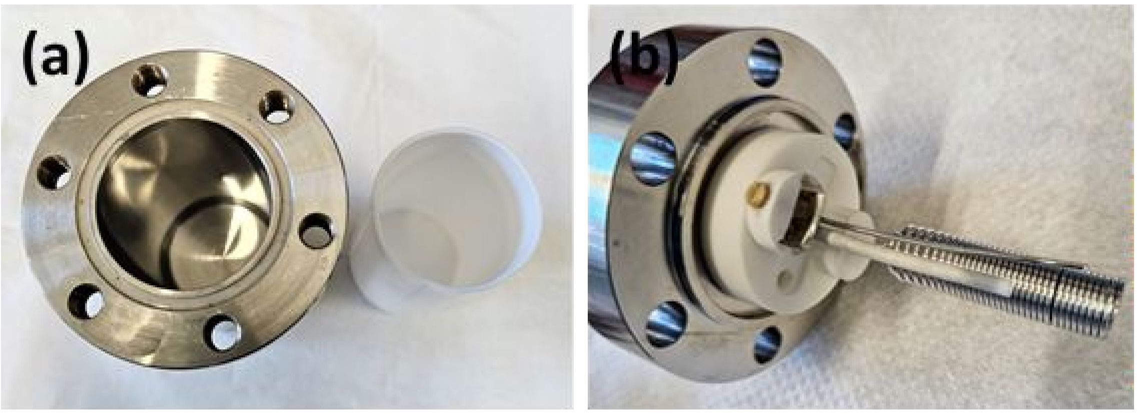


***Figure S2:*** *a) Top view of the autoclave bucket with PEEK sealing and the used Teflon inlet. b) View of the autoclave lid with the Pt coil, the sealed thermocouple, the Pt wire (can be used as pseudo reference electrode), and the clamp to connect the second electrode.*

## 1.3 Electrochemical experiments

Electrochemical measurements were performed with a Gamry Interface 1010E Potentiostat. The experiments were carried out in the electrochemistry autoclave described above in a two-electrode arrangement, i.e. working electrode and counter electrode. If not mentioned otherwise, the Pt coil ($A_{geo}$ ~ 14 $cm^2$), was used as the cathode while the Pt sheet was used as the anode (1 cm x 1 cm). In consequence, the voltage is given as cell voltage, the electrical potential difference between the two electrodes. The anode and cathode chambers are not separated.

In a typical procedure, the autoclave was assembled in a glovebox under inert conditions and filled with the desired pre-dried electrolyte salt. For experiments involving water, the autoclave was sealed outside the glovebox after adding the required amount of MQ water. In this case, the autoclave was flushed multiple times with argon (Ar) to ensure the removal of all other gases. The autoclave was then cooled to 243 K using a refrigerator and subsequently connected to an ammonia bottle to transfer liquefied ammonia ($NH_3$(l)) into the autoclave through the difference in partial pressure between the two vessels. The autoclave was placed on a balance to gravimetrically determine the amount of $NH_3$(l) transferred, with approximately 30 mL transferred for each experiment. After filling, the autoclave was gradually heated to the reaction temperature by a heater, typically 303 K unless otherwise specified. For experiments involving oxygen, ~35 bars of a 4% $O_2$ in Ar mixture were added after the temperature stabilized (Note: *Elevated oxygen partial pressures will eventually result in explosive mixtures if the cathodic hydrogen evolution is not fully suppressed!*[3,4]). The resulting concentration of $O_2$ dissolved in $NH_3$(l) can be estimated based on the works of M. Ahrens and K. E. Heusler, using equation (1):[5]

$$\text{(1)} \qquad \mathrm{c}(O_2) = \frac{p(O_2)}{k} \cdot \frac{\rho(NH_3)}{M(NH_3)} \cdot \frac{1000\,mmol}{mol}$$

| | |
|---|---|
| $\mathrm{c}(O_2)$ | Concentration of $O_2$ dissolved in $NH_3$(l) [mmol $mL^{-1}$] |
| $p(O_2)$ | $O_2$ partial pressure in the cell [Pa] |
| $k$ | Henry`s coefficient [Pa]; $5.09 \cdot 10^9$ Pa |
| $\rho(NH_3)$ | Density of $NH_3$(l) [g $cm^{-3}$] |
| $M(NH_3)$ | Molar mass of $NH_3$ [g $mol^{-1}$] |

Stirring the autoclave during the pressurization step is important, as significant gas dissolution in the liquid ammonia phase was observed. It is therefore crucial to accurately measure the pressure inside the autoclave throughout the pressurization step. As a result, an $O_2$ concentration in $NH_3$(l) of roughly 10 mM was achieved. Subsequently, the autoclave was equilibrated until stable pressure and temperature were achieved, typically taking 10 minutes. Once equilibrated, electrochemical experiments were conducted. First, three cyclic voltammetry (CV) measurements (0–1.5 V; 100 mV/s) were performed to identify the current response and achieve surface equilibration. This was followed by constant current electrolysis to produce the nitrogen oxides. After completing the experiments, the autoclave was placed in a heated water bath (303 K) to carefully remove the gases and evaporate $NH_3$(l) at constant temperature. This is necessary because the autoclave cools down significantly due to the Joule-Thomson effect, which cannot be quickly compensated by the heater. The

remaining solid products were dissolved in 20 mL of MQ water and then analyzed via ion chromatography (IC). After each measurement, the Pt electrodes were cycled 30 times in an aqueous 3 M sulfuric acid solution to restore the surface and prevent fouling by formed $PtN_x$ species.[6] Despite our efforts, the Pt anode did show corrosion and surface oxidation when used for experiments in $NH_3$(l) (Figure S14). Nevertheless, the electrochemical performance of the cell could be maintained.

To calculate the Faraday efficiency (FE) for the production of both nitrite ($NO_2^-$) and nitrate ($NO_3^-$), equation (2) was used:

$$\text{FE}(NO_{2/3}^-) = \frac{F \cdot N(e^-) \cdot n(NO_{2/3}^-)}{C} \cdot 100\% \quad (2)$$

| | |
|---|---|
| $\text{FE}(NO_{2/3}^-)$ | Farraday efficiency for $NO_2^-$ or $NO_3^-$ [%] |
| $F$ | Farraday constant [C $mol^{-1}$] |
| $N(e^-)$ | Electrons needed for the oxidation ($NO_2^-$: 6; $NO_3^-$ : 8) |
| $n(NO_{2/3}^-)$ | Mols of $NO_2^-$ or $NO_3^-$ determined by IC [mol] |
| $C$ | Charge passed [C] |

## 1.4 Gas chromatography (GC)

Headspace analysis was performed by an Agilent gas chromatograph (GC). By using a pressure regulator, the initial pressure was reduced to 1 bar before entering the GC loop. The GC was equipped with a 0.5 m HayeSep Q column, a 1 m Molesieve column and a thermal conductivity detector. Helium was used as carrier gas for $N_2$, $H_2$ and $O_2$ analysis while trace analysis of $H_2$ was performed by switching to Ar carrier gas. For both carrier gases, a flow rate of 22 mL/min was used. Retention time for $H_2$, $O_2$ and $N_2$ are 0.43 min, 0.68 min and 1.16 min, respectively. The column temperature was 323 K.

## 1.5 Infrared spectroscopy (IR)

The gas phase was probed for nitrogen oxides by using gas-phase infrared spectroscopy with a Thermo Scientific Nicolet Avatar 370 FT-IR spectrometer, scanning 42 times per spectrum with a spectral resolution of 2 $cm^{-1}$.

## 1.6 Ion chromatography (IC)

Ion chromatography (IC) measurements were conducted using the Prominence-series liquid chromatography system from Shimadzu. The system was equipped with a CBM-20A controller, an LC-20AD XR pump, a CTO-20AC oven, and an SPD-20A UV/Vis detector set to 218 nm. Separation was achieved using a 250 mm Dionex IonPac™ AS-10 analytical column paired with a 50 mm Dionex IonPac™ AG-10 guard column. The mobile phase consisted of an aqueous mixture of 2.5 mM $Na_2CO_3$ and 1.5 mM $NaHCO_3$, using a flow rate of 1 mL/min (12.3 MPa, 308 K).

In a typical procedure, the sample was diluted by a factor of 5 with MQ water and then directly injected into the ion chromatography system. Injection volume: 2 µL.

Bromide containing samples were subject to an additional precipitation process as large amounts of bromide can influence the quantification of produced nitrate ions. The precipitation was done with a defined volume of a 1.5 M aqueous $Pb(acetate)_2$ solution as precipitating agent. After centrifugation, the supernatant was collected and submitted to the ion chromatography. Blind tests with different nitrite and nitrate concentrations were performed to ensure that additions of $Pb(acetate)_2$ did not affect the experimental results. Additionally, Pb solutions were regularly tested to rule out contamination with nitrite and nitrate.

## 1.7 Raman spectroscopy

Raman investigations of the electrolyte salt were conducted by recording Raman spectra using a 532 nm laser (2.5 mW laser power) on a Renishaw inVia™ REFLEX spectrometer. To investigate the electrolyte salt after the reaction, $NH_3$(l) was carefully evaporated while flushing the autoclave with argon to prevent any moisture from entering the electrochemistry autoclave. Subsequently, the argon-filled autoclave was disassembled in a glovebox. The electrolyte salt was then transferred, in the glovebox, to a transparent sample container to perform the Raman measurements in the absence of air and moisture.

## 1.8 X-Ray photoelectron spectroscopy (XPS)

X-ray photoelectron spectroscopy (XPS) was performed using a custom spectrometer from SPECS GmbH, equipped with a Phoibos 150 hemispherical energy analyzer and a 1D-DLD detector. The monochromatized Al Kα X-ray source (E = 1486.6 eV) operated at 15 kV and 200 W. For high-resolution scans, the pass energy was set to 20 eV, while for survey scans, it was set to 50 eV. The lens mode used was the medium area mode. During the experiment, the base pressure in the analysis chamber was maintained at $3\times10^{-9}$ mbar. Unless otherwise stated, spectra were referenced to the C 1s peak at 284.8 eV to correct for charging effects.

## 1.9 Inductively coupled plasma-optical emission spectrometry (ICP-OES)

Measurements were performed using a Spectro Green FMX 46 ICP-OES. Samples were prepared by dissolving the solids, after ammonia removal (Section 1.3), in MQ water. An 8 mL aliquot of the sample was acidified with 2 mL of nitric acid (65%) and then measured. The resulting signals were quantified using standards obtained from Bernd Kraft.

# 2 Results

## 2.1 Discussion on the reaction mechanism

Based on the results discussed in this study, it is clear that when an external voltage is applied, the reduction of molecular oxygen via the oxygen reduction reaction (ORR) is essential for the formation of nitrite and nitrate, regardless of reaction conditions. The ORR in liquefied ammonia was first studied by Allen Bard and later by Goncalves *et al.*[7–9] In the absence of a proton source during ORR in liquefied ammonia, the first step is reported to be the reduction of $O_2$ via a single electron transfer to the superoxide anion:[9]

$$O_2 + e^- \rightarrow O_2^{\bullet-} \tag{i}$$

Based on the findings of Allen Bard, reaction (i) is quasi-reversible because ammonia is a strongly basic solvent and thus a weak proton donor.[9] It is well known that in organic solvents, the $O_2/O_2^{\bullet-}$ redox pair can be stabilized in polar aprotic solvents.[10] An additional redox feature was observed at more negative potentials than (i) which was attributed to the formation of the peroxide dianion (ii):

$$O_2^{\bullet-} + e^- \rightarrow O_2^{2-} \tag{ii}$$

This feature, was only reversible in the absence of proton sources such as water or ammonium ions.[9] However, the exact origin of this redox feature remains unclear. Studies in aprotic organic solvents have shown that in the presence of weak acids, this redox feature is instead linked to a concerted proton-electron transfer (CPET mechanism; reaction iii), resulting in the formation of the hydroperoxyl radical:[11–13]

$$O_2 + e^- + H^+ \rightarrow HO_2^{\bullet} \tag{iii}$$

In the presence of weak acids, step wise electron-transfer via reaction (i) and subsequent proton-transfer (reaction iv; ET-PT mechanism) can also lead to the formation of the hydroperoxyl radical:[10]

$$O_2^{\bullet-} + H^+ \rightarrow HO_2^{\bullet} \tag{iv}$$

In acidic media, the formation of the hydroperoxyl radical on Pt electrodes is also known to occur through adsorbed hydrogen species on the cathode (Ads. HAT mechanism; reactions v and vi):[10,14,15]

$$O_2 + H^+{}_{ads} + e^- \rightarrow HO_2^{\bullet} \tag{v}$$

$$O_2 + H^{\bullet}{}_{ads} \rightarrow HO_2^{\bullet} \tag{vi}$$

To the best of our knowledge, the mechanism for the ORR in liquefied ammonia has not been investigated in such detail. In the presence of ammonium ions, Goncalves *et al.* found that two electrons are involved in the ORR, which they attributed to the formation of hydrogen peroxide.[8] This finding is typical for the ORR in the presence of protons as the hydroperoxyl radicals eventually decay via a heterogeneous disproportionation to hydrogen peroxide and molecular oxygen (vii):[16]

$$HO_2^{\bullet} + O_2^{\bullet-} \rightarrow HO_2^{-} \quad \text{(vii)}$$

This mechanism has been investigated for DMSO and DMF, and other reactions of the hydroperoxyl radical, such as H-atom abstraction from the solvent (reaction viii), homogeneous disproportionation (reaction ix), or additional ET-PT mechanism (reactions x and xi) might be possible, depending on the reaction conditions, and should therefore be considered.[13,16]

$$HO_2^{\bullet} + SH \rightarrow H_2O_2 + S^{\bullet} \quad \text{(viii)}$$

$$HO_2^{\bullet} + HO_2^{\bullet} \rightarrow H_2O_2 + O_2 \quad \text{(ix)}$$

$$HO_2^{\bullet} + e^{-} \rightarrow HO_2^{-} \quad \text{(x)}$$

$$HO_2^{\bullet} + H^{+} \rightarrow H_2O_2 \quad \text{(xi)}$$

Evaluating the yields of nitrite and nitrate in the presence (with $NH_4Br$ and/or $H_2O$) and absence (with KBr or TBAB) of a proton source suggests that the formation of hydroperoxyl radicals may play a significant role. In the presence of strong proton donors (such as $NH_4Br$), the pronation of the superoxide to the hydroperoxyl radical is diffusion-controlled. However, in the presence of weaker proton donors (like $H_2O$) or aprotic solvents, the protonation of the superoxide anion (reaction iii) is the rate limiting step of the disproportionation.[17] This trend was also observed for the ORR in liquefied ammonia.[8] Based on the findings discussed above, it is plausible that nitrite and nitrate primarily form via in-situ generated peroxide or hydroperoxyl species, as the yields decrease significantly in the absence of $NH_4Br$. However, both studies by Allen Bard[9] and Goncalves *et al.*[7,8] were conducted at 220 and 223 K, respectively. Given the strong influence of temperature on nitrite and nitrate yields (Figure 2), the ORR and the formation of nitrite and nitrate may follow a different mechanism. Notably, increased amounts of non-faradaically formed nitrite and nitrate were observed at temperatures above 303 K (323 K and 343 K). Still, substantially higher yields were observed when an external voltage was applied (see Figure 2 and Figure 4a), leading to FE above 100 %. This could suggest that elevated temperatures may enable a combined mechanism, utilizing both non-faradaic and faradaic oxidation pathways. Also, increased or altered reactivity of the formed ROS under these conditions may influence the overall mechanism. However, this remains largely speculative.

## 2.2 Cell pressure

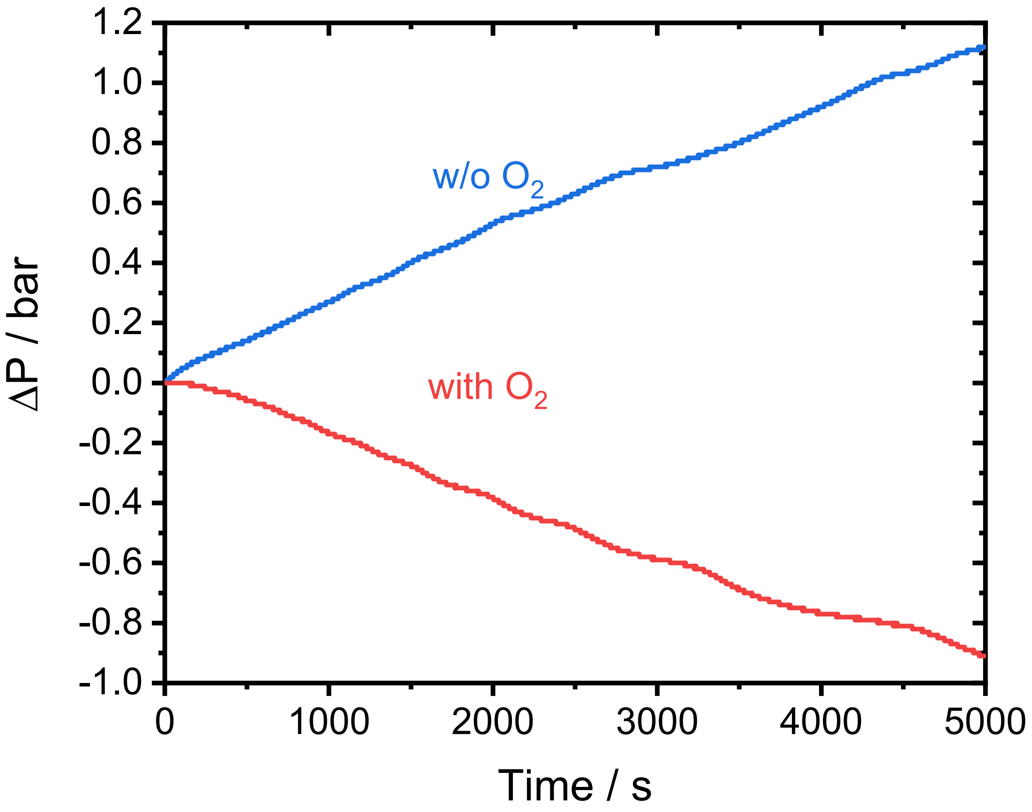


***Figure S3:*** *Pressure of the autoclave in dependence of the reaction time. Reaction conditions: 20 mA currents, 1 M $NH_4Br$ in $NH_3$(l), reaction temperature: 303 K, accumulated charge: 100 C.*

When assuming that the change in pressure is solely attributed to the consumption of $O_2$, the oxygen concentration in the electrolyte can be estimated using equation (1):

$$c(O_2) = \frac{\left(1.4\, bar_{O_2} - 0.9\, bar_{O_2}\right)}{k} \cdot \frac{\rho(NH_3)}{M(NH_3)} \cdot \frac{1000\, mmol}{mol}$$

$$\approx (9.8\, mM - 6.3\, mM) \approx 3.5\, mM$$

According to equation (1), the oxygen concentration dropped by roughly 64% over the course of the experiment. It should be noted that the consumption of oxygen is likely to be higher, as nitrogen evolution at the anode also affects the pressure data in the opposite direction.

## 2.3 Gas phase FT-IR spectra of the headspace

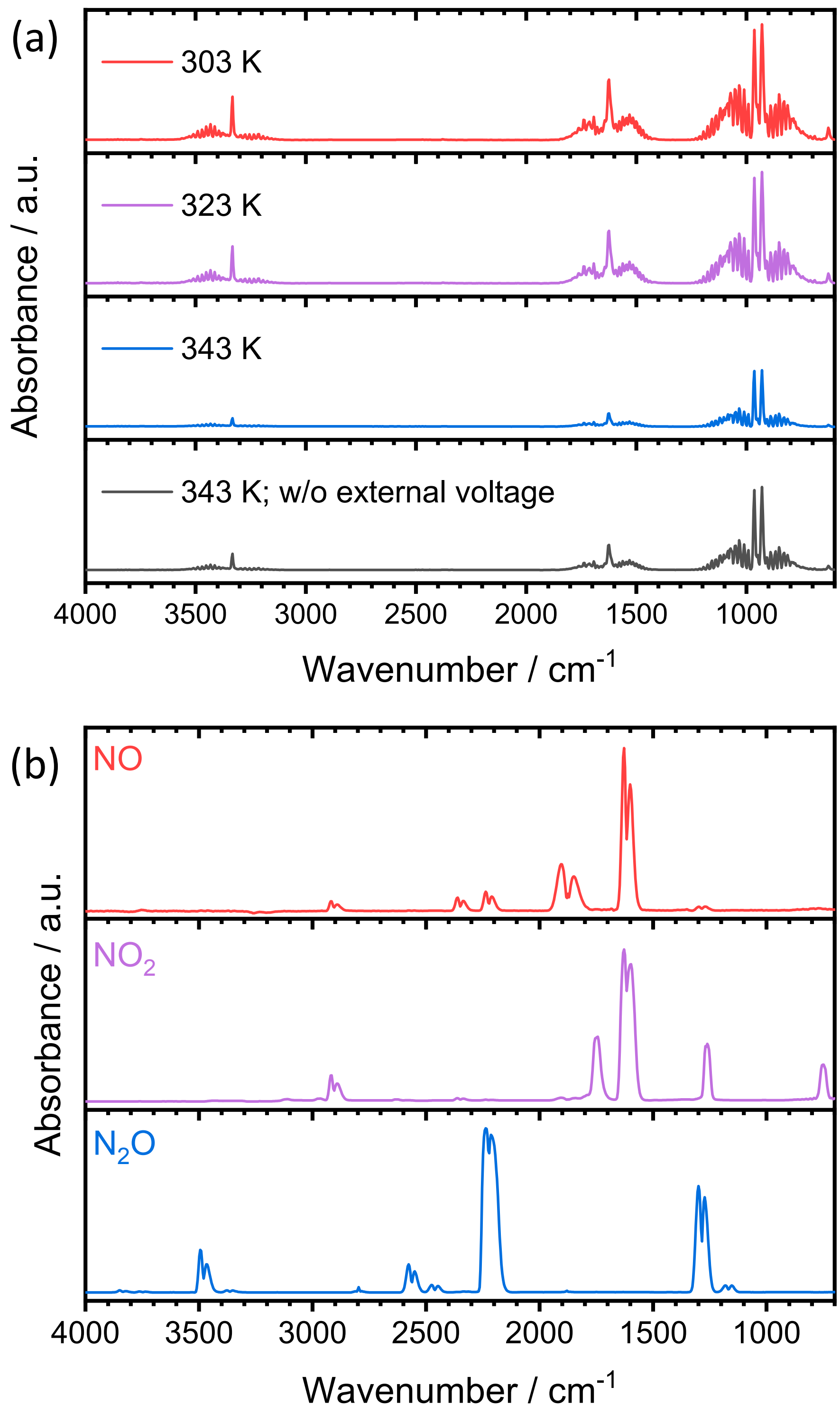


***Figure S4:*** *Gas-phase absorbance FT-IR spectra. (a) Spectra obtained from the headspace of experiments conducted at various reaction temperatures, both with and without applying an external voltage. Reaction conditions: 20 mA, 1 M $NH_4Br$ in $NH_3(l)$, ~35 bars of a 4% $O_2$ in Ar mixture, accumulated charge: 100 C. For the reaction without external voltage, the ammonium containing $NH_3(l)$ was stirred for 3 h at 343 K. (b) Reference spectra collected for different gaseous nitrogen oxides.*

## 2.4 Yields in dependence of the applied currents

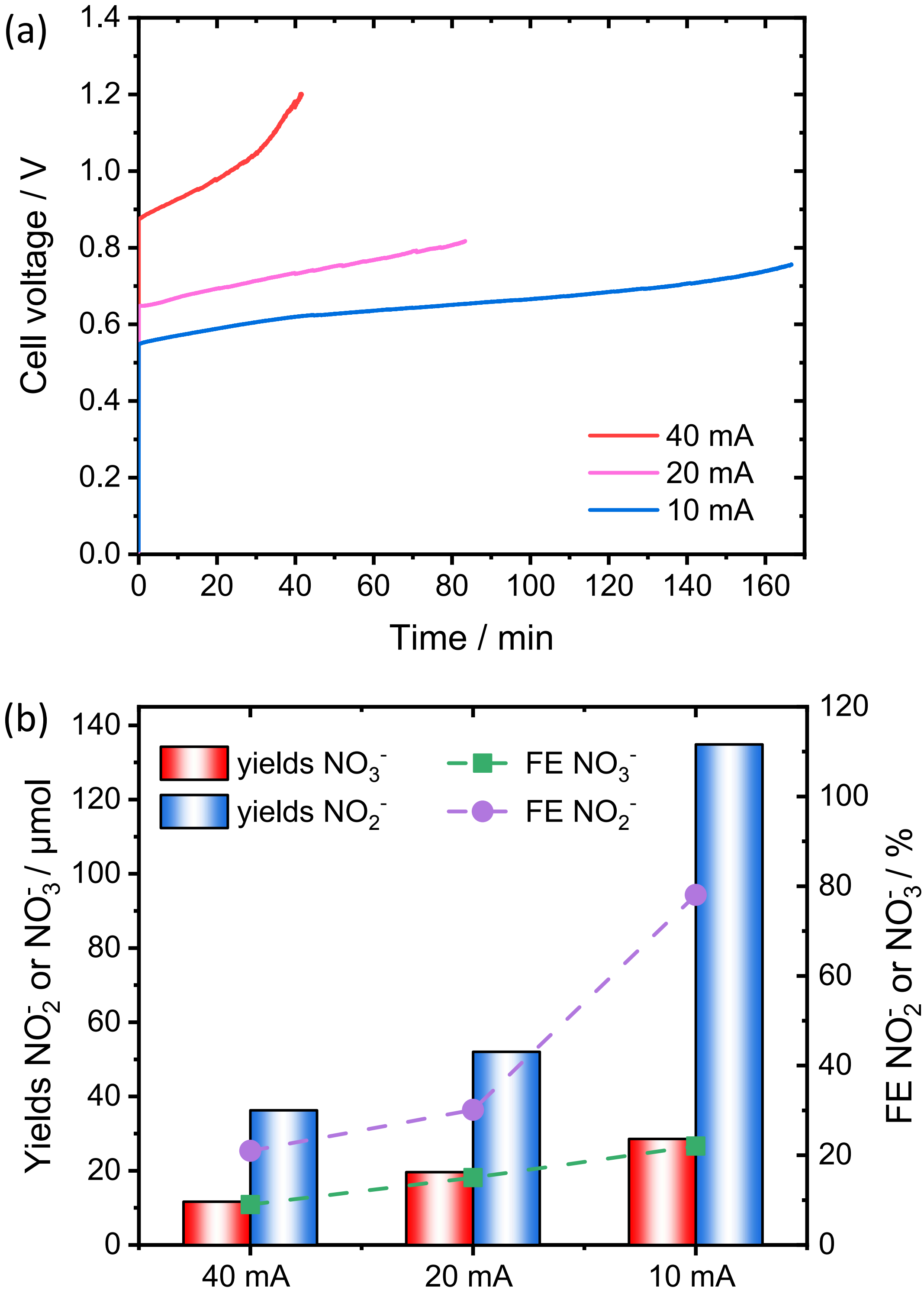


**Figure S5:** (a) Constant current electrolysis at different currents. (b) $NO_{2/3}^-$ yields and FE in dependence of the applied currents during the electrochemical reaction. Reaction conditions: 1 M $NH_4Br$ in NH3(l),, ~35 bars of a 4% $O_2$ in Ar mixture, reaction temperature: 303 K, accumulated charge: 100 C.

## 2.5 Constant current electrolysis at different pressures

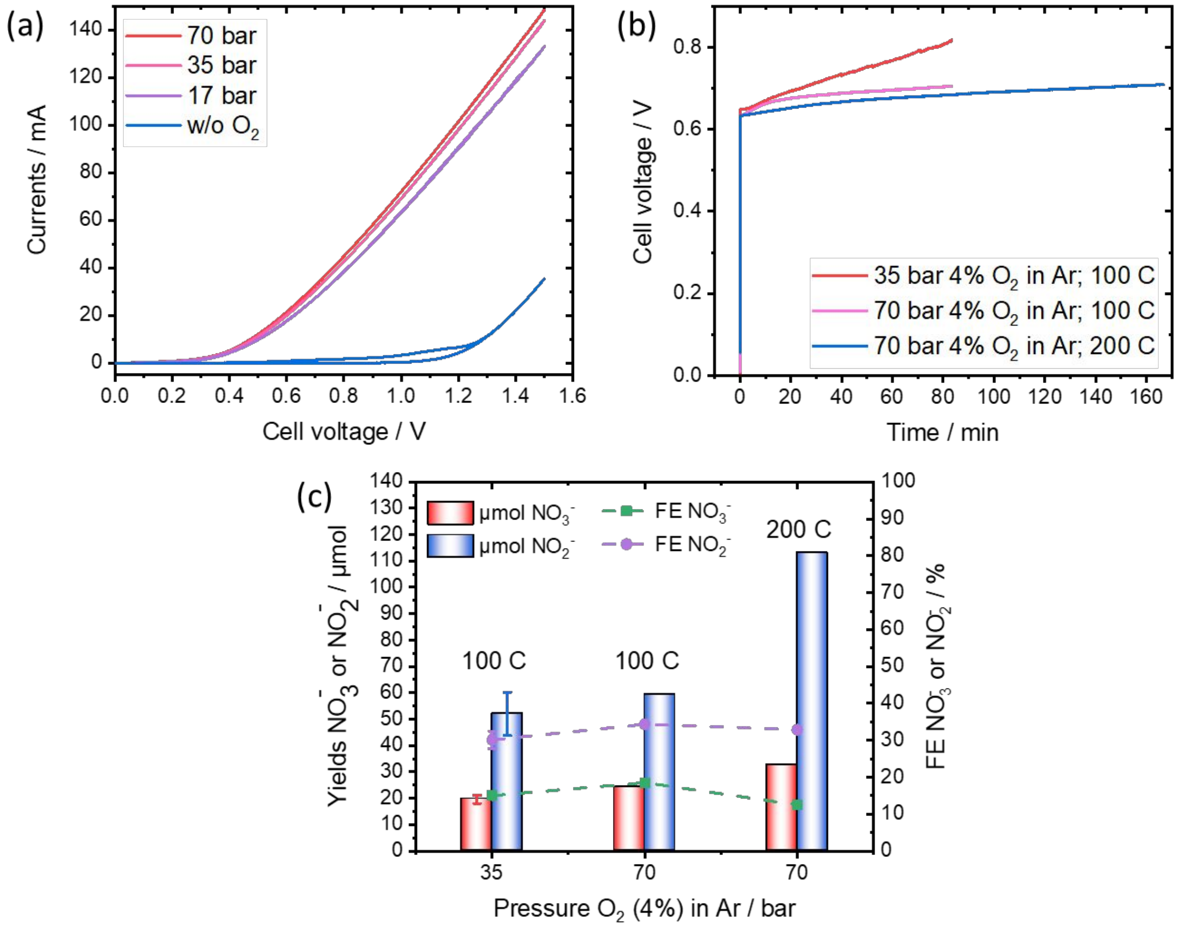


***Figure S6:*** *a) CV measurements at different pressures of the $O_2$(4%) in Ar gas. b) Constant current electrolysis at different pressures. c) Nitrite and nitrate yields from constant current electrolysis (20 mA) at varying pressures and charges. Reaction conditions: 1 M $NH_4Br$ in $NH_3(l)$, 303 K.*

## 2.6 Constant current electrolysis at different temperatures

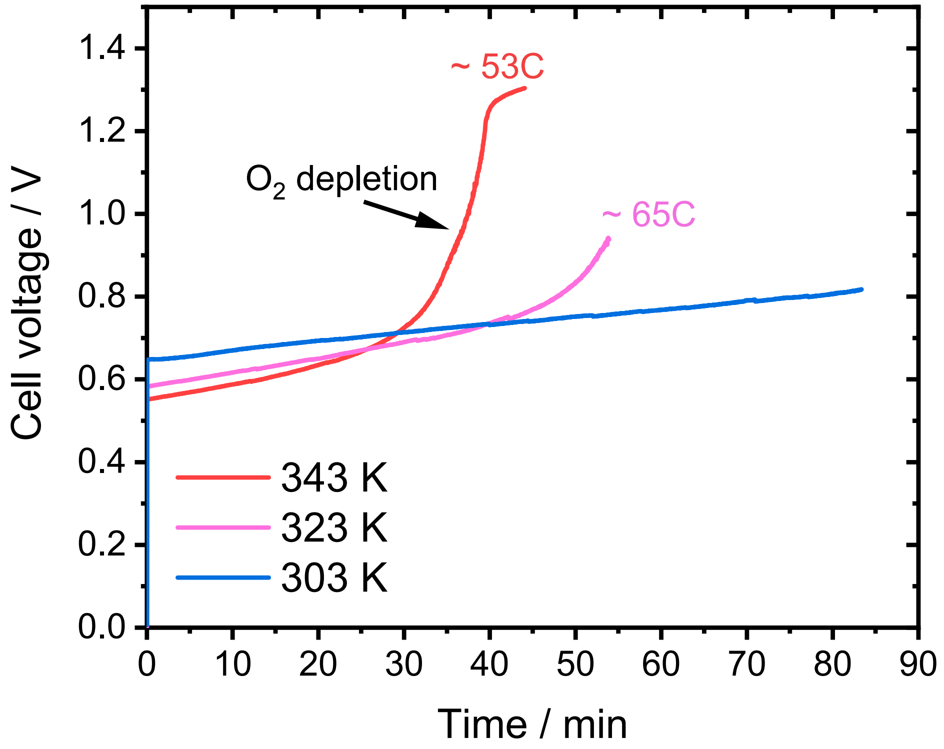


***Figure S7:*** *Constant current electrolysis at different temperatures. Reaction conditions: 20 mA currents, 1 M* $NH_4Br$ *in* $NH_3(l)$*, ~35 bars of a 4%* $O_2$ *in Ar mixture.*

## 2.7 Non-faradaic formation of nitrite and nitrate

To evaluate non-faradaic nitrogen oxide formation as a function of electrolyte salt, blank experiments were conducted by stirring the electrolyte with oxygen (~35 bar $O_2$ (4%) in Ar) for 3 h, followed by ion quantification via IC (Figure S8). The results indicate that $NH_4^+$ promotes non-faradaic nitrite formation, while the absence of $NH_4^+$ results in lower nitrogen oxide yields. Elevated temperatures led to increased nitrite formation even in the absence of $NH_4^+$. Additionally, significant nitrate formation was observed in the presence of $NH_4^+$, which was absent at lower temperatures. These findings demonstrate that nitrite and nitrate formation increases markedly at temperatures above 303 K when the electrolyte is exposed to oxygen for extended periods. However, the time-space yields are significantly lower than those observed when an external potential was applied (see Figure 2 and Figure 4a for comparison).

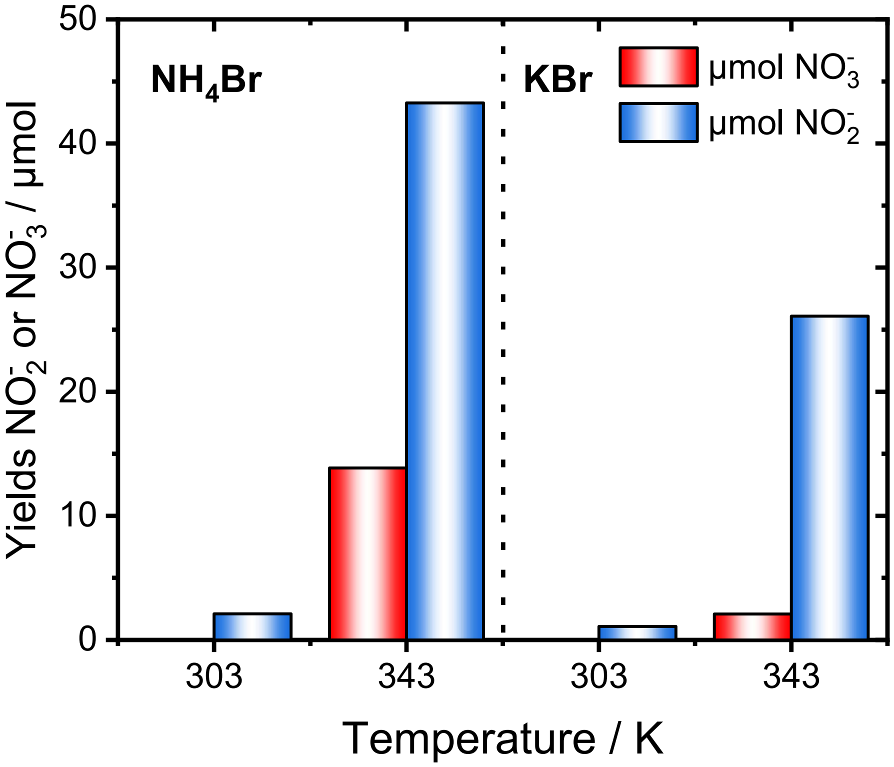


***Figure S8:*** *Yields for nitrite and nitrate obtained when stirring the electrolyte for 3 h in the presence of oxygen without applying an external potential.*

## 2.8 Experiments at different ammonium ion concentrations

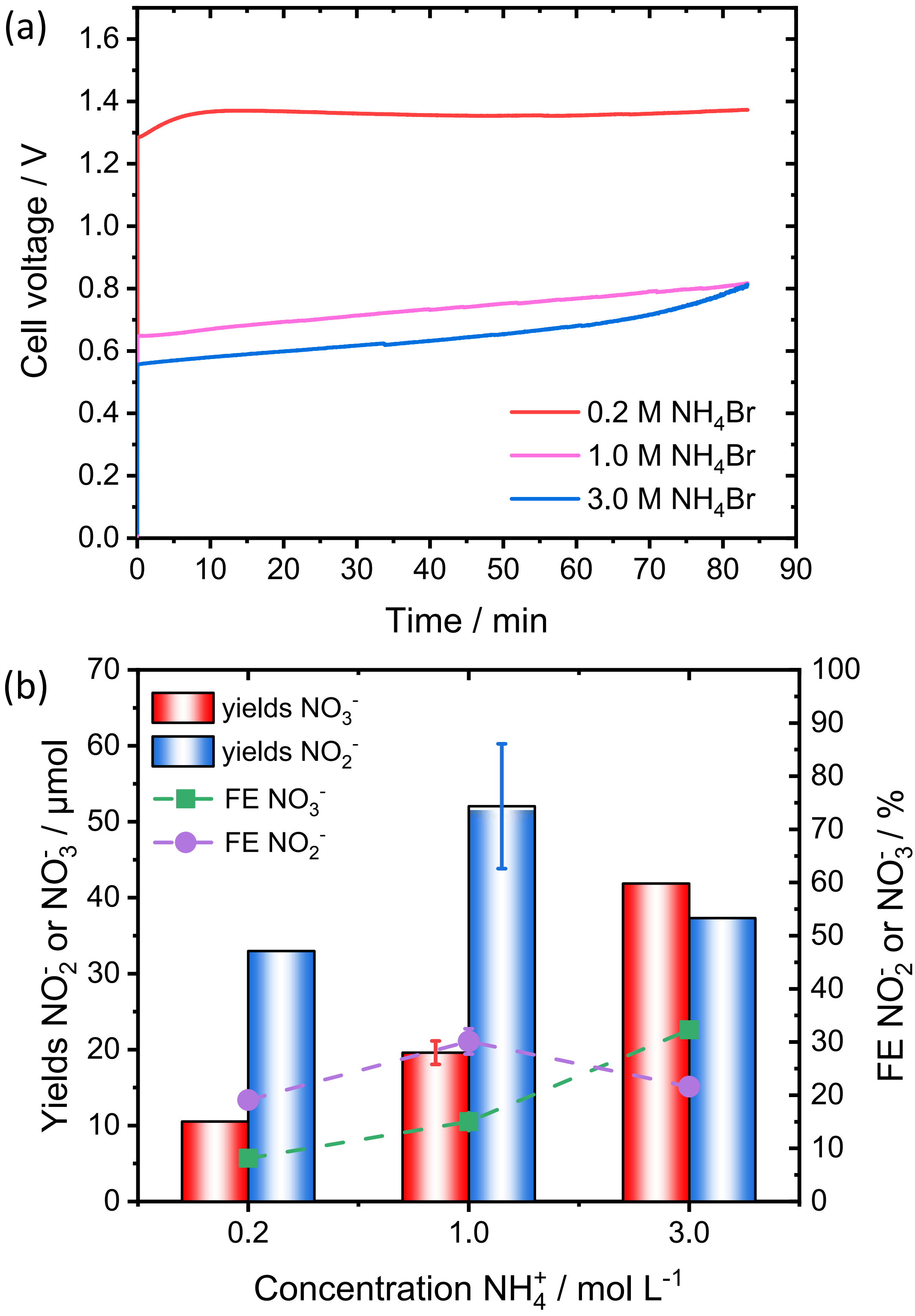


***Figure S9:*** *(a) Constant current electrolysis in the presence of different ammonium ion concentrations. The increase in cell voltage is likely to come from the decreased conductivity of the electrolyte. (b) Corresponding $NO_2^-$/$NO_3^-$ yields and FE. Reaction conditions: Different concentrations of $NH_4Br$ in $NH_3(l)$,, ~35 bars of a 4% $O_2$ in Ar mixture, reaction temperature: 303 K, 20 mA currents, accumulated charge: 100 C.*

## 2.9 Constant current electrolysis in the presence of water

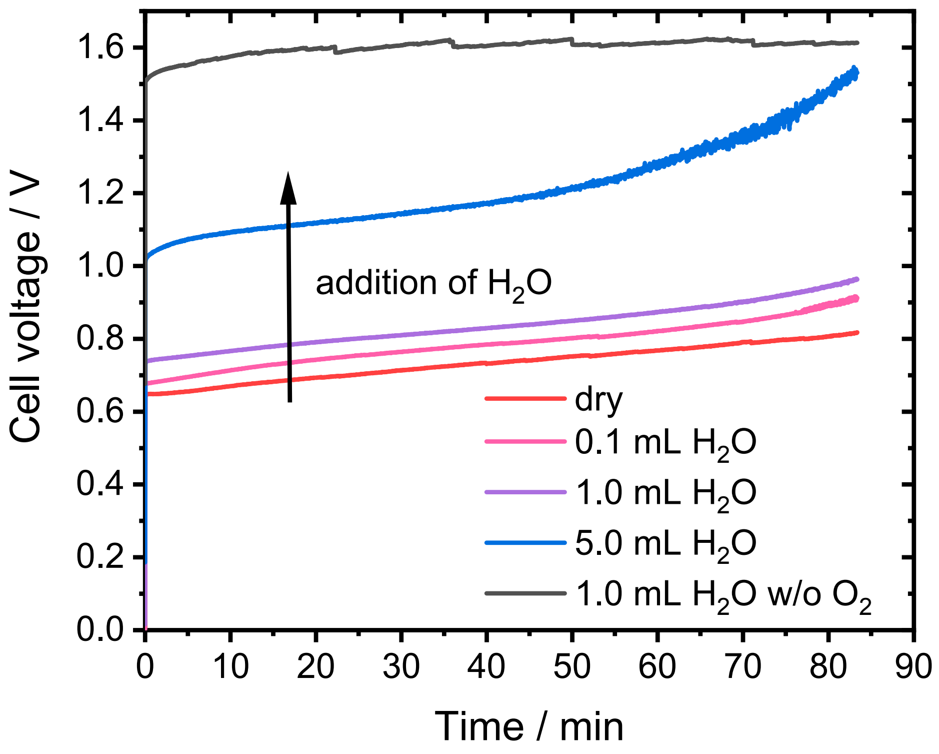


***Figure S10:*** *Constant current electrolysis in the presence of varying volumes of water. Reaction conditions: 1 M $NH_4Br$ in $NH_3$(l), ~35 bars of a 4% $O_2$ in Ar mixture, reaction temperature: 303 K, 20 mA currents, accumulated charge: 100 C.*

## 2.10 Constant current electrolysis for KBr electrolytes

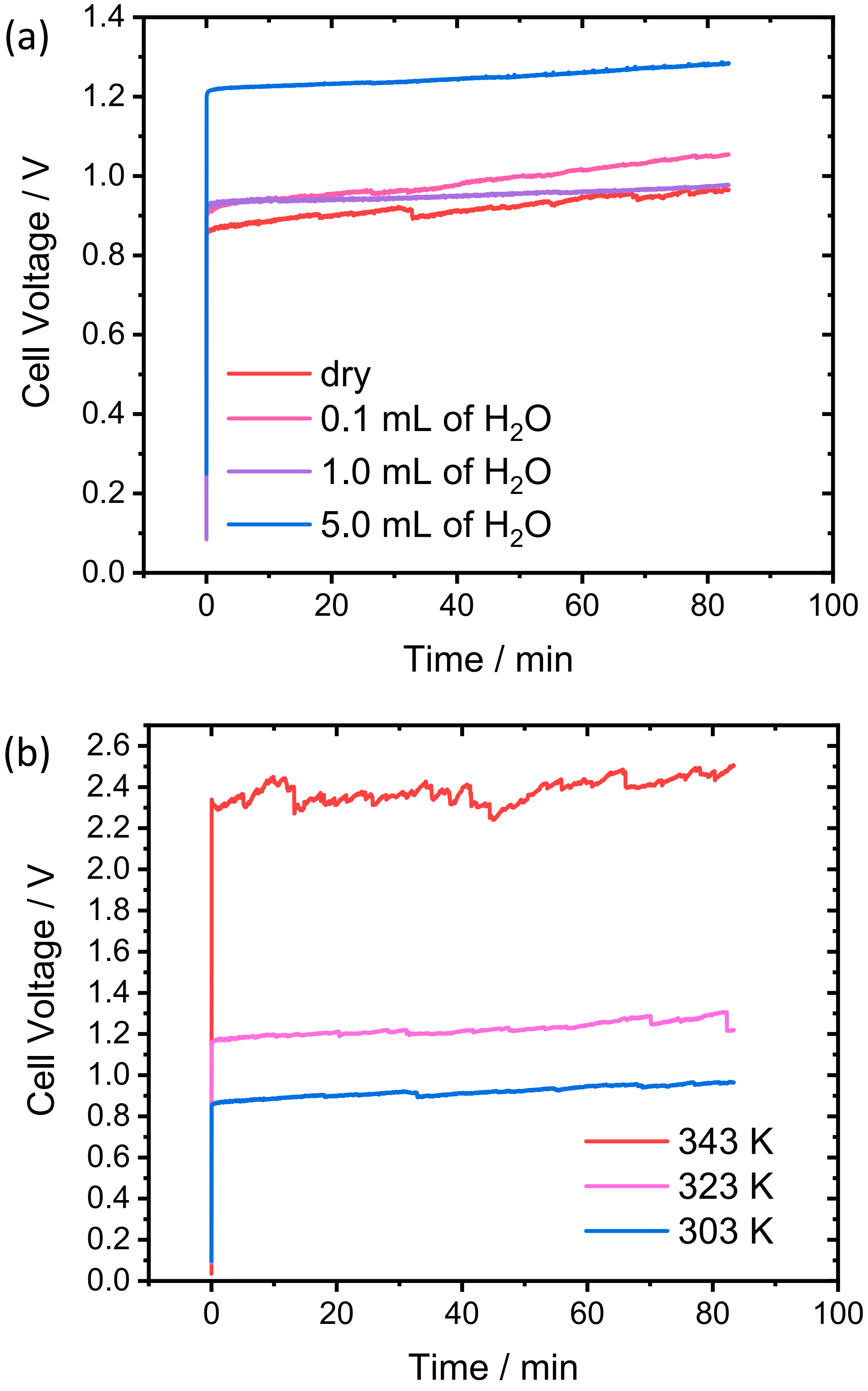


***Figure S11:*** *Constant current electrolysis in the presence of KBr at varying reaction conditions. a) Effect of the reaction temperature on the cell voltage. b) Constant current electrolysis in the presence of water at 303 K. Reaction conditions: 1 M KBr in $NH_3$(l), ~35 bars of a 4% $O_2$ in Ar mixture, 20 mA currents, accumulated charge: 100 C.*

## 2.11 Raman measurements

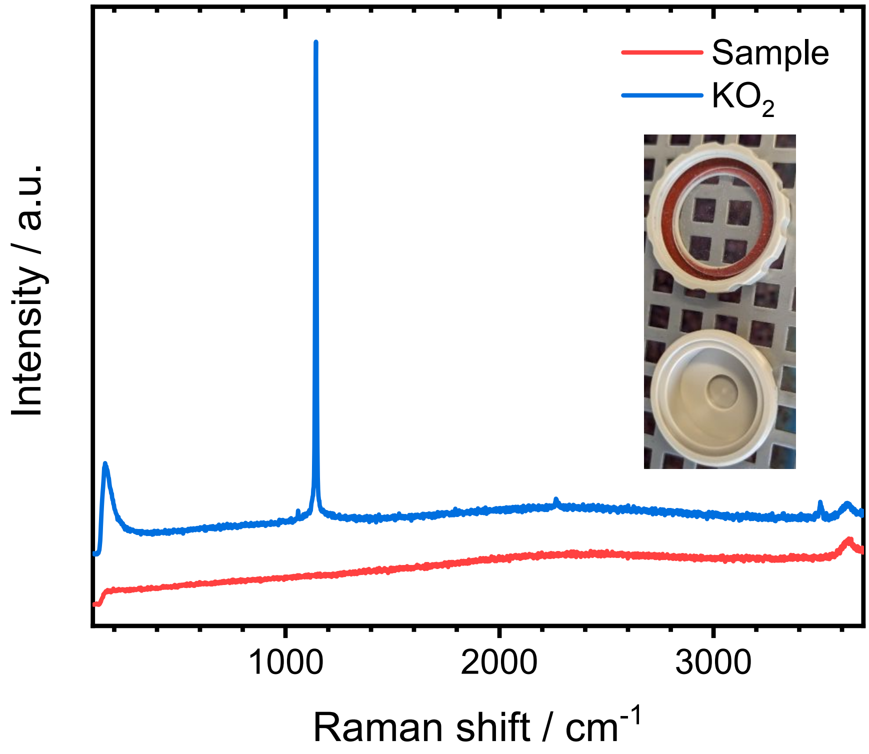


***Figure S12:*** *Raman measurement of the remaining electrolyte salt (KBr) after the electrochemical reaction in the presence oxygen (red). Raman spectra of commercial potassium superoxide ($KO_2$; blue). Insert: Sample holder used for the Raman measurements to prevent contact with air and moisture.*

## 2.12 Experiments with tetrabutylammonium bromide (TBAB)

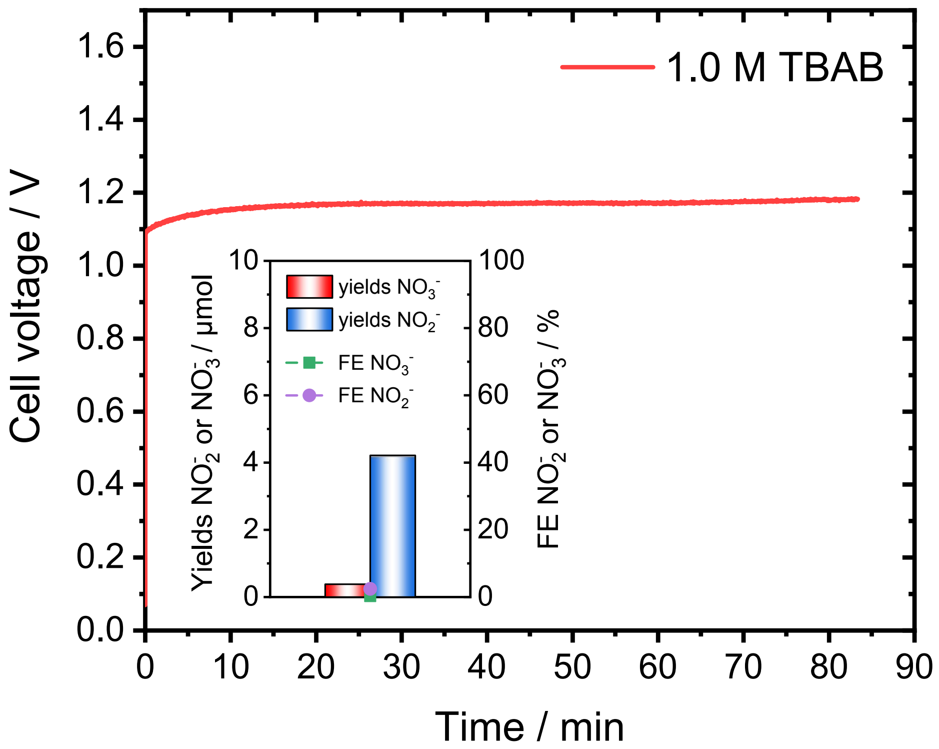


***Figure S13:*** *Constant current electrolysis for experiments using a 1 M TBAB electrolyte. Insert: Corresponding FE and yields for $NO_{2/3}^-$ synthesis when TBAB is used as the electrolyte. Reaction conditions: 1 M TBAB in NH3(l), ~35 bars of a 4% $O_2$ in Ar mixture, reaction temperature: 303 K, 20 mA currents, accumulated charge: 100 C.*

## 2.13 Experiments with ammonium hexafluorophosphate

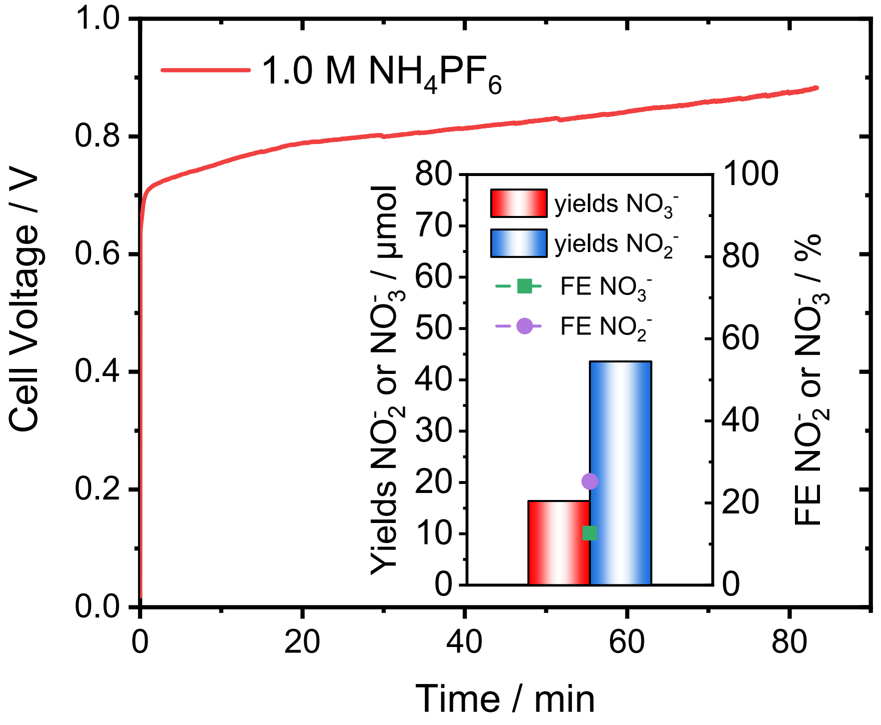


***Figure S14:*** *Constant current electrolysis using $NH_4PF_6$ as the electrolyte salt. Insert: Corresponding FE and yields for $NO_{2/3}^-$ synthesis when $NH_4PF_6$ is used as the electrolyte. Reaction conditions: 1 M $NH_4PF_6$ in $NH_3$(l), ~35 bars of a 4% $O_2$ in Ar mixture, reaction temperature: 303 K, 20 mA currents, accumulated charge: 100 C.*

## 2.14 Experiments in the presence of nitrite and nitrate salts

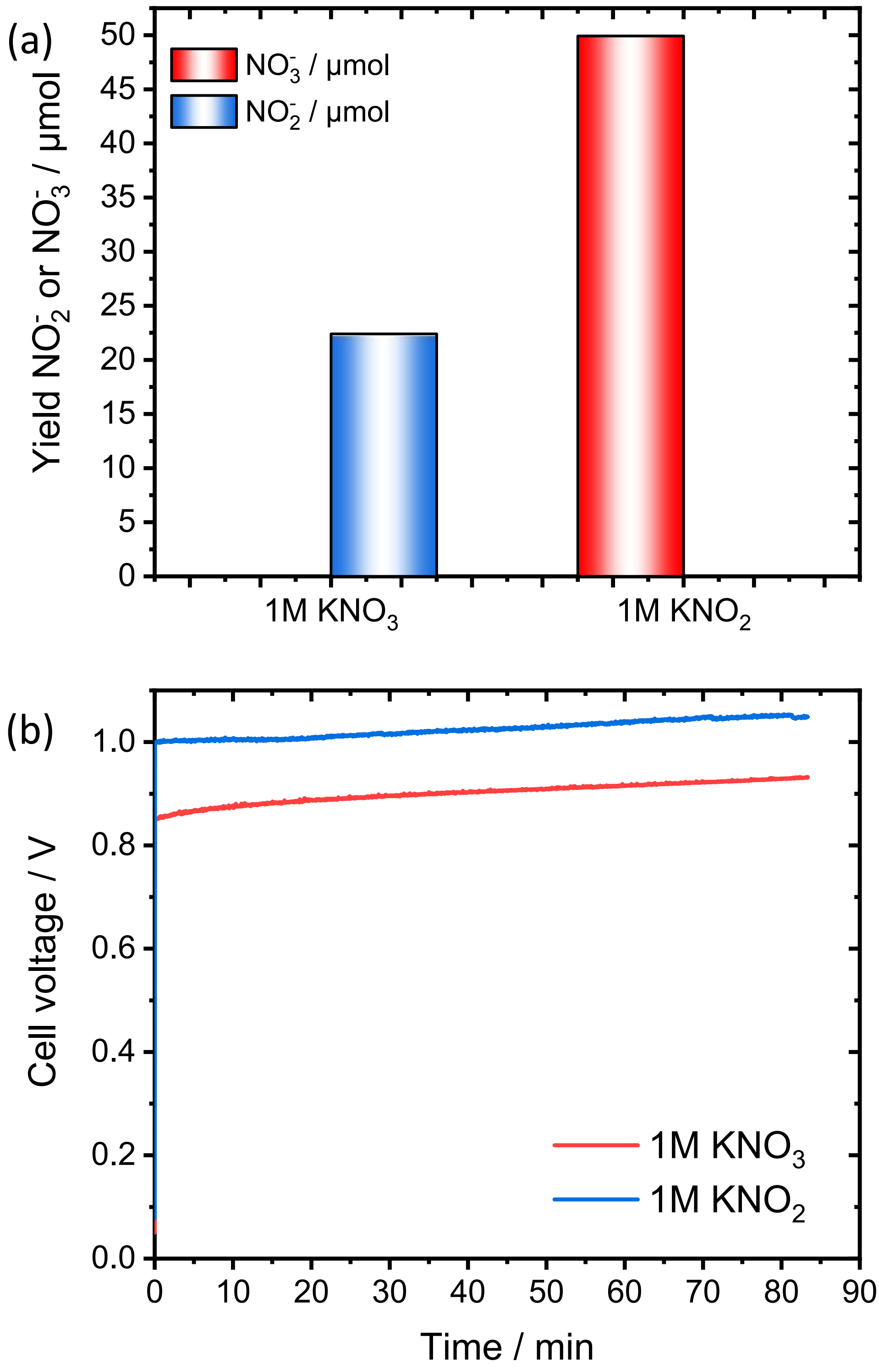


***Figure S15:*** *Experiments performed in the presence of $KNO_2$ and $KNO_3$. a) $NO_2^-$ or $NO_3^-$ yields for the experiments performed with $KNO_3$ or $KNO_2$ respectively. Reaction conditions: ~35 bars of a 4% $O_2$ in Ar mixture, reaction temperature: 303 K, 20 mA currents, accumulated charge: 100 C. b) Corresponding constant current electrolysis.*

## 2.15 Corrosion of the Pt anode

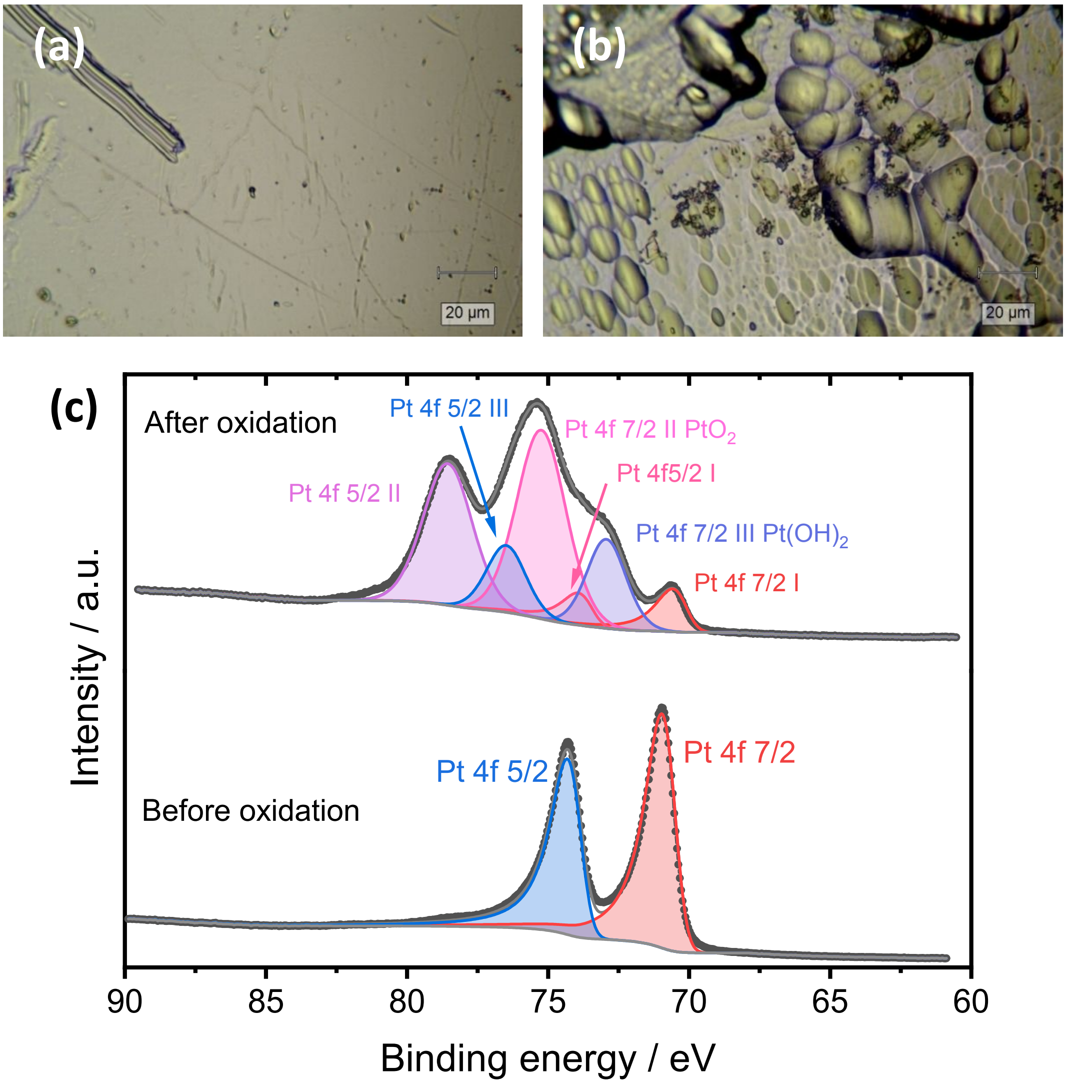


***Figure S16:*** Comparison of the Pt anode before and after the electrochemical reaction. a) Microscope image of a fresh Pt sheet before the reaction. b) Pt surface after the electrochemical reaction, showing leaching and roughening of the Pt surface. c) Pt-XPS spectra a fresh Pt sheet before and after the reaction, revealing surface oxidation of the Pt. It should be noted that the electrode had to be cleaned with water to remove the electrolyte salt. We were not able to oberve any $PtN_x$-species.

***Table S1:*** *Concentrations of dissolved Pt under various conditions. Pt content was determined by ICP-OES.*[a]

| **Experiment** | **Pt leaching / μmol** |
|---|---|
| Pt anode + Pt cathode | 4.42 |
| BDD anode + Pt cathode | 0.26 |
| Control experiment (Pt anode + Pt cathode) | 0.16 |

[a] Pt leaching was quantified after experiments (20 mA, 100 C) using either a combination of a Pt anode and cathode or a BDD anode in combination with a Pt cathode. For the control experiment, identical reaction conditions were applied, except that the electrolyte was stirred without applying an electrical potential for the same duration (5000 s). Reaction conditions: 303 K, 1M $NH_4Br$, ~35 bar $O_2$ (4%) in Ar.

# 3 References

(1) Britschgi, J.; Bilke, M.; Schuhmann, W.; Schüth, F. Indirect Electrooxidation of Methane to Methyl Bisulfate on a Boron-Doped Diamond Electrode. *ChemElectroChem* **2022**, *9* (1), e202101253. DOI: 10.1002/celc.202101253.

(2) Britschgi, J.; Kersten, W.; Waldvogel, S. R.; Schüth, F. Electrochemically Initiated Synthesis of Methanesulfonic Acid. *Angew. Chem. Int. Ed.* **2022**, *61* (41), e202209591. DOI: 10.1002/anie.202209591.

(3) Zhou, C.; Liang, W.; Chen, Z. On Explosion Limits of Ammonia–Oxygen Mixtures with Hydrogen Addition: Sensitivity and Nonmonotonicity. *Energy Fuels* **2021**, *35* (17), 14035–14041. DOI: 10.1021/acs.energyfuels.1c01764.

(4) Schröder, V.; Emonts, B.; Janßen, H.; Schulze, H.-P. Explosion Limits of Hydrogen/Oxygen Mixtures at Initial Pressures up to 200 bar. *Chem Eng & Technol* **2004**, *27* (8), 847–851. DOI: 10.1002/ceat.200403174.

(5) Ahrens, M.; Heusler, K. E. Solubilities of Some Gases in Liquid Ammonia. *Z. Phys. Chem.* **1981**, *125* (1), 127–128. DOI: 10.1524/zpch.1981.125.1.127.

(6) Little, D. J.; Smith, I. M. R.I.I.; Hamann, T. W. Electrolysis of liquid ammonia for hydrogen generation. *Energy Environ. Sci.* **2015**, *8* (9), 2775–2781. DOI: 10.1039/c5ee01840d.

(7) Gonçalves, A.-M.; Mathieu, C.; Herlem, M.; Etcheberry, A. Oxygen reduction mechanisms at p-InP and p-GaAs electrodes in liquid ammonia in neutral buffered medium and acidic media. *Journal of Electroanalytical Chemistry* **1999**, *462* (1), 88–96. DOI: 10.1016/S0022-0728(98)00392-1.

(8) Gonçalves, A.-M.; Mathieu, C.; Herlem, M.; Etcheberry, A. Oxygen Reduction Mechanism in Acidic Liquid Ammonia (223 K): Contribution of Pt Microelectrodes and III-V Semiconductors. *J. Electrochem. Soc.* **2001**, *148* (1), E8–E12. DOI: 10.1149/1.1344544.

(9) Uribe, F. A.; Bard, A. J. Electrochemistry in liquid ammonia. 5. Electroreduction of oxygen. *Inorg. Chem.* **1982**, *21* (8), 3160–3163. DOI: 10.1021/ic00138a048.

(10) Pegis, M. L.; Wise, C. F.; Martin, D. J.; Mayer, J. M. Oxygen Reduction by Homogeneous Molecular Catalysts and Electrocatalysts. *Chem. Rev.* **2018**, *118* (5), 2340–2391. DOI: 10.1021/acs.chemrev.7b00542.

(11) Costentin, C.; Evans, D. H.; Robert, M.; Savéant, J.-M.; Singh, P. S. Electrochemical approach to concerted proton and electron transfers. Reduction of the water-superoxide ion complex. *J. Am. Chem. Soc.* **2005**, *127* (36), 12490–12491. DOI: 10.1021/ja053911n.

(12) Singh, P. S.; Evans, D. H. Study of the electrochemical reduction of dioxygen in acetonitrile in the presence of weak acids. *J. Phys. Chem. B* **2006**, *110* (1), 637–644. DOI: 10.1021/jp055296f.

(13) Savéant, J.-M. Electrochemical Concerted Proton and Electron Transfers. Further Insights in the Reduction Mechanism of Superoxide Ion in the Presence of Water and Other Weak Acids. *J. Phys. Chem. C* **2007**, *111* (7), 2819–2822. DOI: 10.1021/jp068322y.

(14) Sawyer, D. T.; Chiericato, G.; Angelis, C. T.; Nanni, E. J.; Tsuchiya, T. Effects of media and electrode materials on the electrochemical reduction of dioxygen. *Anal. Chem.* **1982**, *54* (11), 1720–1724. DOI: 10.1021/ac00248a014.

(15) Cofré, P.; Sawyer, D. T. Electrochemical reduction of dioxygen to perhydroxyl (HO2.) in aprotic solvents that contain Brønsted acids. *Anal. Chem.* **1986**, *58* (6), 1057–1062. DOI: 10.1021/ac00297a017.
(16) Andrieux, C. P.; Hapiot, P.; Saveant, J. M. Mechanism of superoxide ion disproportionation in aprotic solvents. *J. Am. Chem. Soc.* **1987**, *109* (12), 3768–3775. DOI: 10.1021/ja00246a040.
(17) Chin, D. H.; Chiericato, G.; Nanni, E. J.; Sawyer, D. T. Proton-induced disproportionation of superoxide ion in aprotic media. *J. Am. Chem. Soc.* **1982**, *104* (5), 1296–1299. DOI: 10.1021/ja00369a025.
